# A Church-based Diabetes Self-management Education Program for African Americans With Type 2 Diabetes

**Published:** 2006-06-15

**Authors:** Carmen D Samuel-Hodge, Thomas C Keyserling, Renaé France, Allyson F Ingram, Larry F Johnston, Lisa Pullen Davis, Gwen Davis, Anne S Cole

**Affiliations:** University of North Carolina at Chapel Hill, Department of Nutrition, Schools of Medicine and Public Health; University of North Carolina at Chapel Hill School of Medicine, Chapel Hill, NC; University of North Carolina at Chapel Hill, Center for Health Promotion and Disease Prevention, Chapel Hill, NC; University of North Carolina at Chapel Hill, Center for Health Promotion and Disease Prevention, Chapel Hill, NC; University of North Carolina at Chapel Hill, Center for Health Promotion and Disease Prevention, Chapel Hill, NC; University of North Carolina at Chapel Hill, Center for Health Promotion and Disease Prevention, Chapel Hill, NC; University of North Carolina at Chapel Hill, Center for Health Promotion and Disease Prevention, Chapel Hill, NC; University of North Carolina at Chapel Hill, Center for Health Promotion and Disease Prevention, Chapel Hill, NC

## Abstract

**Introduction:**

Diabetes self-management education interventions in community gathering places have been moderately effective, but very few studies of intervention effectiveness have been conducted among African Americans with type 2 diabetes. This paper describes a church-based diabetes self-management education intervention for African Americans, a randomized controlled trial to evaluate the intervention, and baseline characteristics of study participants.

**Methods:**

A New DAWN: Diabetes Awareness & Wellness Network was conducted among 24 churches of varying size in North Carolina. Each church recruited congregants with type 2 diabetes and designated a diabetes advisor, or peer counselor, to be part of the intervention team. Participants were enrolled at each church and randomized as a unit to either the special intervention or the minimal intervention. The special intervention included one individual counseling visit, twelve group sessions, three postcard messages from the participant's diabetes care provider, and twelve monthly telephone calls from a diabetes advisor. Baseline data included measures of weight, hemoglobin A1c, blood pressure, physical activity, dietary and diabetes self-care practices, and psychosocial factors. The study to evaluate the intervention (from enrollment visit to last follow-up) began in February 2001 and ended in August 2003.

**Results:**

Twenty-four churches (with 201 total participants) were randomized. Sixty-four percent of the participants were women. On average, the participants were aged 59 years and sedentary. They had an average of 12 years of education, had been diagnosed with diabetes for 9 years, had a body mass index of 35, had a hemoglobin A1c level of 7.8%, and had a reported dietary intake of 39% of calories from fat.

**Conclusion:**

A New DAWN is a culturally sensitive, church-based diabetes self-management education program for African Americans with type 2 diabetes that is being evaluated for effectiveness in a randomized controlled trial. The outcomes of A New DAWN will contribute to the literature on community-based interventions for minority populations and help to inform the selection of approaches to improve diabetes care in this population.

## Introduction

Approximately 2.7 million African Americans aged 20 years or older (11.4%) have diabetes, with rates reaching 25% among African Americans aged 65 to 74 and African American women older than 55 (1). Despite the health disparities associated with type 2 diabetes among African Americans compared with whites, relatively few intervention programs have been designed to improve self-management behaviors and metabolic control in this high-risk group. In a recent review of diabetes self-management interventions (2), only one of eight community-based studies published through December 2000 involved African Americans, yet African Americans are almost twice as likely as whites to have diabetes and have two to five times more diabetes complications, including kidney failure, lower limb amputations, and blindness (1). This disparity could be reduced through better self-management behaviors. Diabetes self-management education (DSME) in community gathering places such as churches has been moderately effective and provides opportunities for education that would not normally exist, especially in rural areas (2). Among African Americans, church-based health education and screening programs have effectively facilitated behavior change (3), but only a few reported church-based DSME interventions have targeted African Americans already diagnosed with diabetes (4-5).

We designed a new, culturally sensitive church-based intervention for African Americans with type 2 diabetes to fulfill three main objectives: 1) promote changes in dietary and physical activity behaviors and improve metabolic control, 2) enhance physician-directed outpatient care by providing a community resource to support self-management behaviors, and 3) build churches' capacity to raise the awareness of diabetes' impact on the community's health. The first two objectives are consistent with the model of chronic disease management described by Wagner et al (6), which emphasizes the need for linking the clinical care of patients with diabetes and community support systems for daily disease management. The third objective involves a multilevel intervention — targeting the church as an institution, the community, and the individual. This objective reflects the need for community-based research to integrate opportunities for community capacity building, which would result in communities that have skills and resources at the end of the research period (7,8). This paper describes the intervention A New DAWN: Diabetes Awareness & Wellness Network, the randomized control trial to evaluate the intervention, and baseline characteristics of the churches and study participants.

## Methods

A New DAWN was a year-long church-based intervention comprising four intervention components for participants with diabetes and two intervention components for the entire church membership. The study to evaluate the intervention (from enrollment visit to last follow up) began in February 2001 and ended in August 2003.

### Intervention components for participants with diabetes

**Individual counseling visit**

Each enrolled participant was scheduled for a 60-minute individual counseling session that was held at the church and conducted by a registered dietitian. The counseling visit began with brief assessments of 1) dietary habits (using the food-frequency–based Dietary Risk Assessment) (9); 2) psychosocial issues such as stress management, social support, and problem solving; 3) eating patterns (e.g., timing and consistency of meals); and 4) barriers to dietary and physical activity behavior changes. A detailed description of the assessment, educational materials, and counseling strategies has been published elsewhere (10).

**Group education sessions**

The 12 group sessions held at each church had a basic format and structure. The sequence of sessions was determined by review of standard DSME curricula (11) and a decision to focus on dietary behaviors during the first five sessions. Physical activity and blood glucose self-monitoring were components of each session. Table 1 includes session topics and their associated behavioral and learning objectives. Before each session, participants had their blood glucose level (using the finger-stick method) and blood pressure checked. Participants received feedback on how their blood glucose levels and blood pressure results compared with the American Diabetes Association (ADA) targets and previous entries in the participants' monitoring log. During the monitoring sessions and the group discussions, participants shared successful strategies they had used to make progress toward goals or began brainstorming ways to make progress during the following weeks. After the monitoring was complete, each session opened with a prayer by one of the participants or the church diabetes advisor (CDA) and an overview of the session's content by the group facilitator. The educational component of each session was followed by a short (approximately 15-minute) physical activity segment incorporating chair exercises and a tasting of one or two recipes.

A registered dietitian led the first seven group sessions, with the CDA assisting by greeting the participants, distributing session materials, conducting small group activities, and serving food items for tasting. The educational component of sessions eight through eleven was designed to be led by a health care professional (e.g., pharmacist, dentist, social worker, podiatrist) from the community or church who had been invited by the CDA. This approach was used to 1) teach the CDA how to identify and request the services of community health professionals for church-based activities and 2) expose study participants to the expertise of local health professionals who may not be part of their current diabetes clinical care team. The church was given the option to choose the session topic for the last group session; having a potluck meal was also an option.

When designing the group sessions, we incorporated theories of behavior change and adult education (13,14). Table 2 includes a description of the theoretical basis of the intervention component. We also made sure to design small group activities that were appropriate for individuals with limited literacy or those unaccustomed to group education and interactions. Each session was interactive (nondidactic), included visuals and hands-on activities, required little writing in group activities, used a game format for teaching nutrition concepts whenever feasible, and included opportunities for participants to share their successes and struggles with the group during problem-solving discussions. Because one of our main objectives was to change eating practices through changes in food preparation and selection, we asked church members which dishes were common at church food events, selected recipes for healthier versions of each, and tested the acceptability of all recipes before including them in the group sessions. For each session, two recipes, including a printed copy with nutrient analysis, were prepared for tasting.

We limited the physical activity in each session to chair exercises so that participants with physical activity restrictions could participate and focus on demonstrating activities to improve flexibility and strength. Participants who did not have restrictions on their ability to engage in moderate physical activity were encouraged to engage in 30 minutes of other activity (primarily walking) on most days of the week.

**CDA telephone calls**

When enrolling churches into the trial, we provided each pastor with a flyer describing the personal qualities a CDA should possess and the job-related expectations. After selection by the pastor, the CDAs were trained during 1 month (4 weekly 4-hour sessions) in the areas of motivational interviewing techniques, listening skills, diabetes self-management (with a focus on diet and physical activity), and telephone calls, record keeping, and other administrative responsibilities. The CDA was a peer counselor — a person with type 2 diabetes or who, for at least 2 years, has lived with someone who has diabetes. The CDA contacted participants monthly by telephone and offered support for behavior changes by following up on diet and activity goals, problem solving with the participant, offering information, and providing the participant with community resources.

The first call from the CDA was made after the participant's individual counseling session with the dietitian, during which dietary, physical activity, and diabetes self-care habits were assessed and participants set their first negotiated goals. A copy of these goals and a list of areas in which behavior changes could be made were sent to the CDA to help guide the telephone calls. This assessment was provided as part of the client profile, which included contact information, selected demographic and physiological data, and perceived barriers to diabetes self-care. With the client profile, the CDA received forms to document each telephone conversation, a journal to document any personal information that would help the CDA remember the important issues for each participant, and 12 conversation guides — one for each call. These conversation guides were designed to give the CDA suggestions on how to prepare for and conduct each participant telephone call.

**Postcard messages from health care providers**

DSME programs conducted in community settings are seldom coordinated with participants' clinical care (2). This lack of coordination is a major deficiency in program implementation and an issue we attempted to address with written messages of encouragement from the participants' diabetes care providers. The postcard messages were designed to reinforce the importance of good clinical care and optimal self-management as essential components of diabetes care.

To design the postcard messages, we conducted focus groups with primary care physicians. During these discussions, we evaluated several suggestions for communicating health care providers' support of the feasibility and potential impact of A New DAWN. Although health care providers considered their role as "cheerleaders" of positive behavior changes to be important, they emphasized that they did not want the delivery of these messages to require their time or the time of their staff. Therefore, we developed short, encouraging messages and sent them to participants on the providers' behalf. All messages were fewer than 100 words and addressed general recommendations for physical activity, dietary behaviors, and HbA1c and blood pressure control. We tailored topics to be consistent with goals selected by participants and the overall goals of the study. We sent three postcard messages by mail at approximately 2-month intervals during the first 8 months of the study.

### Intervention components for the church community 

We also designed A New DAWN to offer the entire church community opportunities to become more aware of diabetes prevention and treatment. We used two strategies to accomplish this objective: the Church Health Action Team (CHAT) and church bulletin boards.

#### CHAT

Instead of simply providing diabetes information to churches, we trained a few church members to lead the effort. Each church identified three or four members (along with the CDA) to form a team that would plan and implement churchwide activities to increase diabetes awareness. CHAT members attended a 3-hour workshop in which they were trained to compile materials for a diabetes resource manual and plan a diabetes and health fair.

#### A New DAWN church bulletin board

Diabetes information, recipes from each session, and other program materials were displayed on a bulletin board in a high-traffic area of each church. Displays were changed after each group session.

### Randomized controlled trial evaluation of the intervention

The Figure shows the design of the multisite, randomized controlled trial. After collecting baseline measures, we randomized churches to receive either the special intervention (SI) as described (one individual counseling visit, twelve group sessions, three postcards, and twelve telephone calls) or the minimal intervention (MI). The MI involved a direct mailing to participants of two ADA pamphlets ("Healthy Eating" and "Staying Active") and three bimonthly newsletters that provided general health information and study updates. In addition, each MI church was offered the intervention materials and an opportunity for the study staff members to conduct one group session at the church to show participants how to use the materials. The study protocol was approved by the institutional review board at the University of North Carolina at Chapel Hill. Before enrollment, written informed consent was obtained from each participant.

**Figure 1:**
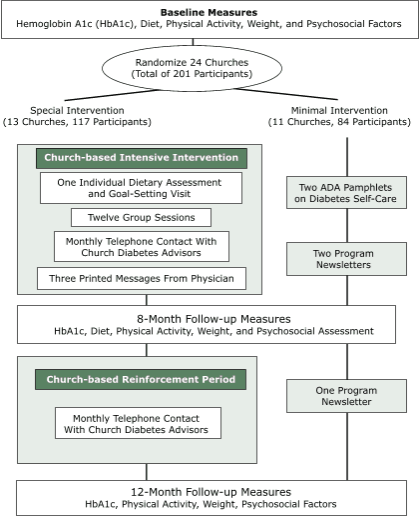
Randomized controlled trial diagram, A New Dawn. ADA indicates American Diabetes Association.

**Table:**

#### Recruiting churches and participants

- **Churches:** Our church recruitment process involved several steps. African American churches of various denominations within 100 miles of the university were identified from a database used by staff members of a previous study (15). Other churches were identified through community contacts and Chamber of Commerce resources. From a listing of more than 300 churches, we contacted 118 churches based on geographical location and congregation size (i.e., 200 or more members). After calling each church, we sent program information to churches interested in the study, and each church was offered the opportunity for a visit by the study staff to fully describe the study and answer questions. The church visit also allowed the study staff to build rapport with the church leaders. After a church decided to participate in A New DAWN, a memorandum of agreement was signed, and plans for participant recruitment began. 
- **Participants:** Each church recruited 8 to 20 members with type 2 diabetes. Inclusion criteria were being aged 20 years or older, having a diagnosis of type 2 diabetes (defined as having a diagnosis of diabetes at 20 years or older and no history of ketoacidosis), having clinical care provided by a primary care clinician, having plans to reside within 50 miles of the church for 1 year, and having a home telephone or easy access to a telephone. Exclusion criteria included having diabetes caused by another condition, being pregnant or lactating, or not being able to speak English.

Our participant recruitment approach incorporated findings from a focus group with pastors and other church leaders. At each church, a liaison was identified to facilitate recruitment, and then the congregation was introduced to the program through posters, pamphlets, and pulpit announcements. Congregants who expressed interest in participating (by calling a toll-free number at the research office, sending a card to the research office, or mentioning their interest to the church liaison) were called by a research assistant, who then assessed eligibility and answered questions about the study. Study staff members scheduled an enrollment visit at the church for each interested person and later conducted three telephone interviews to collect additional baseline data.

#### Study measurements

Physiological measures included HbA1c, weight, and blood pressure. HbA1c was determined from a blood sample collected during enrollment and follow-up visits. Using a standard finger-stick technique, 5 ?l of capillary blood was collected into a capillary collection vial (Bio-Rad, Hercules, Calif), which was stored at 4°C. Blood samples were shipped to the Diabetes Diagnostic Laboratory (University of Missouri, Columbia, Mo), where HbA1c was assessed by automated affinity high-performance liquid chromatography on a Primus CLC-330 system (Primus Corp, Kansas City, Mo). Weight was measured with electronic scales (Seca 770, Seca Corp, Columbia, Md). Blood pressure was measured using the Omron HEM-907 automated blood pressure monitor (Omron Healthcare Inc, Bannockburn, Ill) (16,17). Three blood pressure measures were obtained at 60-second intervals, and the average was recorded.

Physical activity was assessed by the ActiGraph monitor (ActiGraph, Shalimar, Fla), a small, uniaxial accelerometer worn on the waist. Several studies have evaluated the validity of the ActiGraph monitor and report satisfactory correlations between the monitor and other measures of physical activity (18-20).

A food frequency questionnaire (FFQ) administered during the first of three telephone interviews was used to assess dietary intake. We selected the Fred Hutchinson Cancer Research Center 12-page FFQ, an instrument that was previously validated in a sample of women that included African Americans (21).

We measured several psychosocial variables to describe the study sample and evaluate potential determinants of targeted behavior changes. (The results will be presented in a future article.) Among the 15 psychosocial constructs measured, 10 were developed or adapted for an African American population from previously validated measures; all new or adapted measures have demonstrated acceptable psychometric properties. Psychosocial measures included church involvement and spirituality, coping styles (22), diabetes and general health status (23,24), perceived diabetes competence (25), perceived stress (26), diabetes problem areas (27) and perceived social and diabetes-related barriers (28), social support (29), familial roles (30), self-efficacy (31), and stages of dietary and physical activity behavior change (32). Most measures were administered during two telephone interviews; the exceptions were measures of perceived stress level and stages of dietary and physical activity behavior change, which were administered in person at the enrollment visit.

#### Sample size, randomization, and statistical methods

Sample size calculations were based on the following assumptions for the primary study outcome, which was the comparison of HbA1c levels between treatment groups at the 8-month follow-up: a two-sided test (α = 0.05); a power of 80% to detect a mean difference of 1% in HbA1c levels between groups; a cluster (church) randomization with an intraclass correlation (ICC) of 0.031 (based on findings in a previous study) (33); and 10 participants per church. Twenty-eight churches (280 participants) were required. However, we were only able to recruit 24 churches and 201 participants. Consequently, with an average of eight participants per church, an observed intraclass correlation for the baseline HbA1c of 0.0064, expected attrition of 10%, and assumptions as noted, our power to detect a 1% difference in HbA1c between groups was 72%.

To prevent contamination between the SI and MI interventions, we randomized each church as a unit. Randomization was stratified by church location (rural or nonrural, as defined by the United States Postal Service) because of presumed location differences in church size, church members, and organization. Using computer-generated random numbers, a statistical consultant prepared a set of sequentially numbered sealed envelopes containing study group assignments. Churches were assigned to a study group after required baseline data (dietary, physical activity, and demographic information) were collected from all participants within a church.

Comparisons of baseline characteristics between study groups controlled for randomization by church were made using mixed-effects models (34). This approach adjusts for correlation among observations from the participants within each church. For skewed non-Gaussian data, we used the Mann-Whitney U test for clustered data (35). In each model, the baseline characteristic being compared was the dependent variable; the intervention group was treated as a fixed effect, and the church was treated as a random effect. SAS software (SAS Institute, Cary, NC) was used for the analysis, and all reported *P* values are two sided.

## Results

Of 118 churches initially contacted, 30 agreed to participate, and of those, 24 enrolled the required minimum number of participants. The 24 churches were randomized to the SI or MI group — 13 churches (117 participants) to the SI group and 11 churches (84 participants) to the MI group. Comparisons of selected characteristics of churches by intervention group assignment (i.e., SI or MI) revealed no significant group differences according to denomination (e.g., Baptist vs non-Baptist), number or age distribution of active members, age or education of pastor, or previous (i.e., in past 2 years) participation in research projects.

Baseline characteristics of participants are shown in Table 3. All but 1.5% (n = 3) of participants identified themselves as African American (data not shown). On average, participants were 59 years of age, had been diagnosed with diabetes for 9 years, and had completed the 12th grade; 64% were women. Approximately half of the participants were married or living with a partner and were currently employed; 45% had total household incomes of less than $30,000, and 8% had no health insurance. Although only about 8% of participants were current smokers, 75% were being treated for hypertension, and 19% had known coronary heart disease. Self-reported dietary intake showed that 39% of total calories came from fat, with 12% coming from saturated fat. The mean HbA1c was 7.8%, and the mean body mass index (BMI) was 35. Overall, participants were sedentary and participated in minimal minutes per day of moderate or vigorous physical activity.

Information on participants' diabetes-related medical care and self-care behaviors is presented in Table 4. Although participants reported having frequent appointments with their diabetes physician (an average of 4.6 appointments per year), only 17% had received dietary counseling on two or more occasions during the previous year. Approximately 75% of participants were taking oral diabetes medications, 29% were taking insulin, and 12% were taking no diabetes medication. Almost all (96%) participants had been screened for high blood cholesterol, with 33% taking cholesterol-lowering medication and 50% taking aspirin regularly. No significant differences were found between groups.

Although many reported consuming a low-cholesterol and low-fat diet, less than a third said they were following a diet to lose weight or following a diet for diabetes on 5 to 6 days per week (data not shown). Approximately 85% of participants measured their blood glucose levels at home; about 75% did so once per day. Overall, participants in both treatment groups were similar, although the SI participants were significantly younger.

## Discussion

A New DAWN is a church-based DSME intervention designed to be culturally appropriate and consistent with current behavioral and educational approaches for diabetes self-management. This intervention not only includes culturally sensitive features in design, delivery, and evaluation but also incorporates features that address the psychosocial and contextually relevant influences on health behaviors (36,37). Although they are not well-defined in the literature, we believe the latter factors are reflected in A New DAWN’s focus on 1) organizing education around the “church family” (with family members included as study participants and invited as guests to the group sessions); 2) life experiences as a source of experiential knowledge through the CDA and group interactions and sharing during sessions; 3) using CDAs as cultural translators and diabetes advocates; and 4) measuring psychosocial factors using instruments that were developed and tested for cultural relevance and linguistic appropriateness. Special attention was given to the conceptualization and measurement of psychosocial factors in this group. Surveys were developed or adapted for cultural and linguistic appropriateness and tested for construct validity. A New DAWN was designed and implemented with the collaboration of researchers and the church community, making it a culturally relevant intervention capable of producing useful contextual information (38) to enhance DSME research in this setting.

Only one other church-based DSME intervention for African Americans tested by a randomized controlled trial design has been published (5), so A New DAWN will provide important information related to the design, implementation, and effectiveness of church-based diabetes interventions. The Patient-Owned Enhanced Education Regimen (POWEER) intervention (5) was conducted in four large urban churches (with 109 participants) in the northern United States. In contrast, results from A New DAWN (with 201 participants) will provide information about a southern, more rural population in 24 churches of varying membership size. In addition, results from both trials will allow evaluation of different aspects of the church environment on program acceptability and outcomes. It may be important to have information on how programs designed with the church as a social unit, with existing social relationships among participants who share a common church experience (A New DAWN), differ from those with the church as a community gathering place for intervention activities (POWEER). Data from POWEER indicate that attendance for the intervention group sessions was best among participants recruited from the church (5).

A review of A New DAWN's design and the sample of enrolled churches reveals some limitations as well as potential applications. First, it was challenging to recruit churches into the trial — only 25% of churches contacted agreed to participate, and 20% of those who agreed were not randomized because they did not recruit enough participants, pastors changed their minds about participation, or leadership changed. Some reasons that churches may not have wanted to participate include 1) not wanting to add a new program because of numerous existing outreach efforts (39); 2) lack of positive experiences with research participation (with only 22% of our sample having participated in research projects during the last 2 years); and 3) considering diabetes to be a medical condition best handled by health care professionals in a clinical setting. If the church is to play an important role in supporting self-management behaviors for African Americans (within the framework of chronic disease management), then the success of programs such as A New DAWN may increase the community’s awareness of how churches can play a role in improving diabetes care. Second, the role of the CDA in interventions such as A New DAWN will need additional evaluation to assess its potential in facilitating church support systems for patients with diabetes.

Participants recruited from churches for A New DAWN differed in important ways from participants in other clinic-based diabetes interventions (10,33,40) conducted in central North Carolina. A larger proportion of A New DAWN participants had private health insurance, and the participants had higher educational levels and higher incomes than participants in studies by Rothman et al (40) and Keyserling et al (10,33). Moreover, A New DAWN participants were less likely to be using insulin when they enrolled, and they had lower HbA1c levels than participants in the other studies. The baseline characteristics that were similar among participants in all of these studies include age, sex, duration of diabetes, and BMI.

The characteristics of participants who volunteered for A New DAWN limit how much we can generalize the study outcomes to other groups of African Americans. Other limitations include 1) a study design that does not allow for evaluation of specific program components on outcome variables (e.g., the independent effects of the CDA or physician messages on metabolic or behavioral changes) and 2) outcome measures that focus primarily on people with diabetes, so the impact of intervention components such as the CHAT, church bulletin board, and CDA on behaviors and awareness of diabetes among church members are not adequately assessed.

To address the high prevalence of diabetes among African Americans, more research is needed to develop effective community-based programs. Eliminating the health disparities associated with diabetes requires culturally appropriate and effective interventions that address the individual's role in self-management and the community's role in supporting self-care. In an attempt to meet this need for effective interventions, we developed A New DAWN as a culturally appropriate program that incorporates salient aspects of the chronic disease model (6). The outcomes of A New DAWN will contribute to the literature on community-based interventions for minority populations and help to inform the selection of approaches to improve diabetes care in this population.

## Figures and Tables

**Table 1:** Group Sessions and Associated Learning and Behavioral Objectives, A New DAWN

**Session^a^**	**Title**	**Learning and Behavioral Objectives**
1	What Is Diabetes? Living With Diabetes	To review basic diabetes information (risk factors, causes and symptoms of high and low blood glucose, and key factors in living well with diabetes) To introduce study goals and group session format
2	What's in Your Food: Carbohydrates, Protein, and Fat. Knowing Your Serving Sizes	To group foods by their major macronutrients and relate carbohydrate content to glycemic effect To enhance awareness of portions served and serving sizes
3	Healthy Eating — Fiber and Fat	To discuss and encourage use of blood glucose self-monitoring logs To introduce relationship between blood glucose and hemoglobin A1c (HbA1c) values To review recommendations for healthy eating, with a focus on fiber and fats
4	Planning Meals — Plate Method	To introduce plate method and other strategies for planning meals To provide menus (15 days) and review how they could be used for better diabetes control
5	Shopping and Eating Out	To practice reading the nutrition facts on food labels and review nutrient claims (e.g., low fat, light, low sodium) To increase awareness of fast foods' nutrient and caloric content
6	Blood Glucose Self-Monitoring	To review importance of blood glucose self-monitoring, target blood glucose ranges, and recommended HbA1c values To increase knowledge of how food and physical activity affect blood glucose levels
7	Blood Pressure Control	To review the meaning of blood pressure numbers, recommended blood pressure levels for people with diabetes, and importance of blood pressure control in protecting kidney health To increase knowledge of how diet and physical activity affect blood pressure levels (with focus on DASH eating pattern and taking 10,000 steps per day)
8	Diabetes Medications	To introduce pharmacist as knowledgeable community member and medication expert To discuss how different diabetes medications work and provide participants with opportunity to ask questions about medications they are currently taking (not only diabetes medications)
9	Personal Health Habits 1^b^	To introduce podiatrist as knowledgeable community member To review good health habits related to skin and foot care and make connection between smoking and foot health To increase knowledge of Medicare coverage of therapeutic footwear for people with diabetes
10	Personal Health Habits 2^b^	To introduce dentist as knowledgeable community member To increase awareness of how diabetes affects dental health, including proper denture care
11	Stress Management	To introduce a community health professional who is knowledgeable of strategies to reduce stress and improve mental well-being To discuss how dealing with stress can help with diabetes self-management
12	Church's Option^c^	To review progress made toward reaching goals set in A New DAWN program To offer an opportunity for program participants to interact and celebrate their accomplishments

DASH indicates Dietary Approaches to Stop Hypertension (12).

a Opportunities for self-monitoring blood glucose and blood pressure, physical activity, and tastings were behavioral components of each session.

b Personal health habits include foot, skin, and dental care.

c Church members decide on session topic or to have social event with potluck.

**Table 2:** Theoretical Basis for A New DAWN Intervention Components

**Theory and Construct**	**Intervention Component**	**Intervention Strategies**
**Social cognitive theory**		
*Environment:* external physical factors that influence behavior	Church-based group education Church diabetes advisor (CDA)	Select the church as comfortable and friendly environment that will facilitate learning. Implement program in participants' community church. Groups at each church are made up of congregants, family, and friends; family and friends are invited as guests to group sessions. Have participants interact with CDA by home telephone.
*Situation:* perception of the environment	Group sessions	Begin each session with a prayer (following format of other church meetings by including a spiritual component). Post general diabetes information, session topics, and recipes on A New DAWN bulletin board in church to raise awareness of diabetes.
*Behavioral capacity:* knowledge and skills to perform a behavior	Group sessions CDA telephone calls	Include in group sessions opportunities to learn and practice ways to decrease saturated fat and sugar in food preparation, include activity in daily living, manage stress, get social support, and solve problems. Include in each session a short physical activity segment and tasting of recipes or new foods. CDAs share information and successful strategies for diabetes self-management during telephone calls.
*Observational learning:* learning by watching actions of similar others	Group sessions CDAs	Community members (health professionals, such as dentists, pharmacists, podiatrists) lead selected group sessions. CDAs act as role models and group cofacilitators. (CDAs are church and community members with diabetes.)
*Reinforcements:* behavior responses that increase or decrease likelihood or reoccurrence	Physician Monthly goal setting CDA telephone calls	Have physicians recognize performance of patients and provide encouragement and feedback; use printed physician messages as source of encouragement. Give self-selected rewards for reaching goals as component of monthly goal setting. During telephone calls, have CDAs give praise and other forms of social support for positive behavior changes.
*Self-efficacy:* confidence in performing a behavior	Health counselor assessment and tailoring Group sessions CDA	Use short-term goal setting with small, achievable steps to facilitate changes in diet and physical activity behaviors; record on log sheets progress in increasing physical activity and controlling blood pressure and blood glucose. Share successes in changing behavior during sessions and with CDA during telephone calls. Perform different types of physical activity during group session. Use scenarios and hands-on activities to practice skills in making dietary and other behavioral changes.
**Stages of change model**	New Leaf Diabetes educational materials Health counselor assessment and tailoring	Use assessments and tip sheets to target different stages of readiness for behavior change. Have the health counselor develop individually tailored action plans using assessment data and input of participant; identify and address attitudinal barriers using brief assessments and corresponding tips for making behavior changes.
**Adult learning theory**	Group sessions	Have participants provide input on topics for group sessions. Use group discussions to encourage sharing of life experiences to facilitate learning and behavior change.
**Social networks and social support**	Church and community setting Group sessions CDA Physician	Invite families and friends to group sessions. Use church members as a community support unit. Have CDA make monthly telephone calls to enhance social support.Use printed physician messages to add social support value.
**Community health development**	Church and community setting CDA and Church Health Action Team (CHAT)	Solicit community input in development and implementation of intervention. CDA gains employment and develops skills in health communication, diabetes self-management, and behavior change strategies through training for position and cofacilitator role in group sessions. CHAT develops community resource list and plans church events to raise awareness of diabetes; CHAT learns skills in planning church health events through participation in planning workshop.

**Table 3:** Baseline Characteristics of A New DAWN Participants, by Intervention Group

**Characteristic**	**SIN = 117**	**MIN = 84**	***P* Value^a^**
**Demographics**			
Age, y, mean (SE)	57.0 (0.9)	61.3 (1.3)	.007
Female, %	64.2	63.1	.87
Years with diagnosed diabetes, mean (SE)	8.8 (0.8)	9.2 (0.9)	.77
Years of educational achievement, mean (SE)	12.6 (0.4)	12.2 (0.5)	.48
Currently living with spouse or someone like a spouse, %	48.5	57.9	.29
No. of adults (counting self) ≥18 y living at home, mean (SE)	2.0 (0.08)	2.1 (0.06)	.30
No. of children <18 y living at home, mean (SE)	0.5 (0.08)	0.4 (0.05)	.20
Currently employed, %	48.1	45.7	.74
Unemployed (n = 61 [SI]; n = 45 [MI]), %			
Retired, %	83.5	79.8	.63
Disabled, %	54.2	54.5	.97
Total annual household income <$30,000 (n = 120), %	44.5	45.0	.96
Health insurance (uninsured),^b^ %	8.0	8.6	.86
**Risk factors for coronary disease, %**			
Current cigarette smoker	10.1	6.7	.50
Currently being treated for high blood pressure	73.9	76.1	.82
Positive family history for coronary heart disease^c^	26.1	23.0	.47
Known coronary heart disease^d^	18.9	18.8	.98
**Dietary recall data, mean (SE)**			
Total calories	1349 (54.8)	1220 (61.7)	.12
Cholesterol (mg/dL)	245 (14.1)	214 (18.1)	.18
Macronutrient consumption, % of calories			
Carbohydrate	47.0 (1.3)	45.4 (1.3)	.40
Protein	16.4 (0.34)	16.4 (0.40)	.91
Total fat	38.2 (1.1)	39.5 (0.86)	.37
Monounsaturated fat	14.8 (0.47)	15.5 (0.42)	.28
Polyunsaturated fat	8.9 (0.27)	8.8 (0.17)	.77
Saturated fat	11.6 (0.31)	12.1 (0.28)	.29
Trans fat	2.6 (0.06)	2.5 (0.10)	.35
**Hemoglobin A1c (HbA1c), body weight, and blood pressure, mean (SE)**			
HbA1c	7.7 (0.2)	7.9 (0.3)	.12
Weight, lb	213 (4.3)	216 (5.8)	.69
Body mass index	34.6 (0.7)	35.1 (0.8)	.62
Systolic blood pressure	139 (1.7)	140 (2.2)	.60
Diastolic blood pressure	75 (0.6)	76 (1.2)	.37
**Physical activity,^e^ %**	**N = 104**	**N = 76**	** **
No. of hours monitor worn per day	12.2 (0.2)	12.5 (0.2)	.29
Total days monitor worn	6.2 (0.1)	6.5 (0.1)	.034
Light activity (<3.0 METs) min/day, median (IQR)	45.3 (19.8-74.07)	44.9 (23.6-72.0)	.57
Moderate activity (3.0-5.9 METs) min/day, median (IQR)	5.6 (1.7-11.4)	3.8 (1.8-10.1)	.91
Vigorous activity (6.0-8.9 METs) min/day, median (IQR)	0 (0.0-0.0)	0 (0.0-0.0)	.52

SI indicates special intervention; MI, minimal intervention; MET, metabolic equivalent; IQR, interquartile range.

a All data and comparisons between groups are adjusted for study design (randomization of churches to SI or MI).

b Percentages equal greater than 100% because some participants had more than one type of health insurance.

c Physician diagnosis of myocardial infarction or angina or history of percutaneous intervention for coronary heart disease or coronary artery bypass surgery.

d Myocardial infarction or sudden death of unexplained cause in father or brother before age 55 years or mother or sister before age 65 years.

e For minutes of physical activity per day, medians and IQR are crude data; however, *P* values for these comparisons are adjusted for study design (randomization by church).

**Table 4:** Diabetes-Related Clinical Care by Intervention Group, A New DAWN

**Characteristic**	**SI (N = 117)**	**MI (N = 84)**	***P* Value**
Number of visits per year with diabetes physician, mean (SE)	4.8 (0.3)	4.3 (0.5)	.42
Counseled by dietitian 2 or more times in previous year, %	20.6	13.7	.27
Ever had blood cholesterol checked, %	95.5	96.2	.78
When cholesterol was checked, was it high?, % (N = 104 for SI; 74 for MI)	42.2	26.8	.055
Dilated eye examination in last year, %	77.3	73.8	.62
Dental examination in last year, %	57.2	46.0	.05
Flu vaccine in last year, %	53.3	50.9	.76
Diabetes treatment, %			
Oral medications	70.9	78.6	.24
Insulin	32.0	25.2	.36
Any diabetes medication	83.8	91.5	.10
Currently taking cholesterol-lowering medication, %	31.6	33.4	.78
Currently taking aspirin three or more times weekly, %	48.7	50.8	.70
Currently taking hormone replacement therapy, %	22.6	26.3	.59

SI indicates special intervention; MI, minimal intervention.
